# Meta-analysis of expression and methylation signatures indicates a stress-related epigenetic mechanism in multiple neuropsychiatric disorders

**DOI:** 10.1038/s41398-018-0358-5

**Published:** 2019-01-22

**Authors:** Kaiyi Zhu, Tai-Hsien Ou Yang, Vincent Dorie, Tian Zheng, Dimitris Anastassiou

**Affiliations:** 1 Department of Systems Biology, Columbia University, New York, NY 10032 USA; 2 Department of Electrical Engineering, Columbia University, New York, NY 10027 USA; 3 Data Science Institute, Columbia University, New York, NY 10027 USA; 4 Department of Statistics, Columbia University, New York, NY 10027 USA; 5 Present Address: Roche Innovation Center, New York, NY 10016 USA

## Abstract

Similar environmental risk factors have been implicated in different neuropsychiatric disorders (including major psychiatric and neurodegenerative diseases), indicating the existence of common epigenetic mechanisms underlying the pathogenesis shared by different illnesses. To investigate such commonality, we applied an unsupervised computational approach identifying several consensus co-expression and co-methylation signatures from a data cohort of postmortem prefrontal cortex (PFC) samples from individuals with six different neuropsychiatric disorders—schizophrenia, bipolar disorder, major depression, alcoholism, Alzheimer’s and Parkinson’s—as well as healthy controls. Among our results, we identified a pair of strongly interrelated co-expression and co-methylation (E–M) signatures showing consistent and significant disease association in multiple types of disorders. This E–M signature was enriched for interneuron markers, and we further demonstrated that it is unlikely for this enrichment to be due to varying subpopulation abundance of normal interneurons across samples. Moreover, gene set enrichment analysis revealed overrepresentation of stress-related biological processes in this E–M signature. Our integrative analysis of expression and methylation profiles, therefore, suggests a stress-related epigenetic mechanism in the brain, which could be associated with the pathogenesis of multiple neuropsychiatric diseases.

## Introduction

Major psychiatric disorders, such as schizophrenia and bipolar disorder, and neurodegenerative diseases, such as Alzheimer’s and Parkinson’s, are all pathologically related to abnormalities in the brain^1–4^ with different manifestations in each case, but the underlying etiologies remain largely elusive. Extensive research has implicated environmental factors in the pathogenesis of such neuropsychiatric disorders^5–9^, operating through epigenetic mechanisms to change gene expression and thereby disrupting particular biological functions in brains. For example, in the case of several types of dementia, including Alzheimer’s disease, environmental influences have been associated with the risk of disorder, which can lead to epigenetic transformations, such as altering DNA methylation and histone modification, over time^10^. Furthermore, striking experimental evidence connecting environmental stress and pathogenic outcome has been provided by research on both animals^11–13^ (rodents and primates) and humans^14,15^ (monozygotic twins with discordant disease states). The LEARn (latent early-life associated regulation) model was proposed^16^ as an epigenetic explanation for neurobiological disorders. On the other hand, it has been shown that transcriptomes and DNA methylation patterns of different brain regions differ substantially^17,18^ across brain regions. In this study, we focused on one specific brain region, the prefrontal cortex (PFC), which has been implicated in the etiology of different neuropsychiatric diseases^19,20^.

We reasoned that the co-expression and co-methylation modules can be used as signatures to represent some particular biomolecular events, and that the modules shared by different diseases indicate common mechanisms. Moreover, analyzing the interrelationships between signatures can help in further understanding such mechanisms. For example, a significant association observed between some co-expression and co-methylation signatures can indicate a particular epigenetic regulation.

For that purpose, we first assembled multiple publicly available gene expression and DNA methylation data sets obtained from postmortem adult PFC samples across six different neuropsychiatric disorders along with healthy controls. We then identified a number of “consensus” PFC co-expression and co-methylation signatures, present in similar forms across multiple data sets, using an unsupervised methodology^21^. Integrative analysis of these signatures along with supervised analysis of available phenotypic associations suggested that a particular epigenetic abnormality could be involved in the pathogenesis of different neuropsychiatric illnesses.

## Materials and methods

### Data sets and preprocessing

Our discovery data cohorts consist of publicly available expression data and DNA methylation data of postmortem PFC samples from 426 subjects (242 cases, 184 controls) and 823 subjects (406 cases, 417 controls), respectively. The details of sample information can be found in Table 1. Most of the publicly available human postmortem PFC samples were obtained using the Affymetrix Human Genome U133 array and the Illumina HumanMethylation 450k beadchip for expression and DNA methylation, respectively. Because our multi-data-set algorithmic implementation works best with uniform data properties, we restricted the data sets to those types of profiling platform. We eliminated from consideration data sets representing repeated runs of the samples from the same subjects, thus avoiding replicates in the data cohorts that we collected for consensus signature identification, which could have otherwise biased the results. We also required that the number of either cases or controls should be at least 10 for expression data and 15 for methylation data (because of the larger number of probes for the methylation platform).

**Table 1:** Description of data sets assembled for consensus analysis. (a) Gene expression cohorts. (b) DNA methylation cohorts

(a)				
Data set	Disease	Platform	Brain region	Sample size (control:case)
Ryan et al.^55^ [GSE5388]	Bipolar disorder	HG-U133A	BA9	31:30
Maycox et al.^56^ [GSE17612]	Schizophrenia	HG-U133-P2	BA10	23:28
Zhang et al.^57^; Zheng et al.^58^ [GSE20168]	Parkinson’s	HG-U133A	BA9	15:14
Narayan et al.^59^ [GSE21138]	Schizophrenia	HG-U133-P2	BA46	29:30
SMRI AltarC	Multiple^a^	HG-U133A	BA46/10	11:33
SMRI Bahn	Multiple^b^	HG-U133A	BA46	33:65
Chang et al.^60^ [GSE54567/54568/54570]	Major depression	HG-U133A	BA9	42:42

(b)				
Data set	Disease	Platform	Brain region	Sample size (control:case)
Xu et al.^25^ [GSE49393]	Alcoholism	HM450	BA9	23:23
Lunnon et al.^61^ [GSE59685]	Alzheimer’s	HM450	PFC	24:56
Wockner et al.^62^ [GSE61107]	Schizophrenia	HM450	PFC	24:24
Pidsley et al.^63^ [GSE61380/61431]	Schizophrenia	HM450	BA9	38:38
Jaffe et al.^64^ [GSE74193]	Schizophrenia	HM450	BA46/9	240:191
Smith et al.^65^ [GSE80970]	Alzheimer’s	HM450	PFC	68:74

*BA* Brodmann area, *HM450* Illumina Infinium Human Methylation 450 Beadchip, *PFC* prefrontal cortex (for which Brodmann areas not specified), *HG-U133A* Affymetrix Human Genome U133A Array, *HG-U133-P2* Affymetrix Human Genome U133 Plus 2.0 Array, *SMRI* Stanley Medical Research Institute

a. Cases consist of 11 bipolar disorder, 11 major depressive disorder, and 11 schizophrenia patients

b. Cases consist of 31 bipolar disorder and 34 schizophrenia patients

We downloaded the raw data sets and preprocessed them as follows. Data sets with gene-expression values were profiled using either of two Affymetrix platforms, but we only used HG-U133A probes for analysis in this study so that every individual expression data set contains the same probe set. The raw CEL files were log-transformed and RMA normalized for each individual data set with default settings as implemented in the R Bioconductor *affy* package^22^. For DNA methylation analysis, we obtained *β* values from the methylated and unmethylated signal intensities for each individual data set by using the *dasen* function in the R Bioconductor *wateRmelon* package^23^.

The validation data sets used in this study include additional microarray and RNA-seq data sets. The microarray data sets include GSE36980 for Alzheimer’s disease^24^ and GSE49376 for Alcoholism^25^, which were not included in the consensus analysis because they were profiled on different platforms. We normalized them in the same way as we did for the Affymetrix microarray data. The RNA-seq data sets, covering four out of the six neuropsychiatric diseases, include PFC samples from GSE68719 for Parkinson’s^26^, GSE101521 for major depression^27^, bipolar disorder and schizophrenia samples as part of the BrainGVEX study (available on Synapse with accession number syn4590909) within the PsychENCODE Consortium^28^. For RNA-seq data sets deposited on Gene Expression Omnibus (GEO), we normalized the raw counts individually using DESeq2^29^, removed genes whose expression values were zero in more than half of the samples, and then performed log2-transformation. For the BrainGVEX data, we downloaded the normalized version from https://github.com/mgandal/Shared-molecular-neuropathology-across-major-psychiatric-disorders-parallels-polygenic-overlap/tree/master/working_data/RNAseq.

### Attractor-finding algorithm

#### General version for individual data sets

The attractor-finding algorithm is an unsupervised method for identifying signatures of mutually associated features from a matrix containing values of features (rows) in different samples (columns). Therefore, using expression or methylation matrices, it identifies co-expression and co-methylation signatures, respectively.

The details of the general attractor-finding algorithm can be found in our previous work^21,30^. Briefly, the algorithm uses an iterative procedure to collect mutually associated features, converging to the core (“heart”) of the underlying co-expression or co-methylation mechanism. The association measure we used is based on the mutual information (MI)^31^, which generally captures even nonlinear relationships between variables. To outline the process in the case of gene expression data, it starts from a “seed” (e.g., the expression of one particular gene). In the first iteration, all genes are ranked in terms of their MI with the seed gene, and a “metagene” is created as a hypothetical gene whose expression values, for each sample, are equal to the weighted average of the expression values of all genes, where each weight is defined as a function of the MI of that gene with the seed gene. Each subsequent iteration updates the metagene, so that the weight of each gene in the new metagene is defined as a function of the MI of that gene with the previous metagene. The process is repeated until convergence to an “attractor metagene”. From the attractor metagene, we can extract the top-ranked genes (those with the highest weights), together with a “score” (ranging from 0 to 1) for each of these genes, which measures the “strength” of the membership of that gene in the signature. If the strength of, say, the 10th ranked gene is >0.5, this suggests that there is a strong co-expression involving at least ten genes, and that the genes with the highest scores in the attractor metagene point to the core of the biological mechanism underlying that co-expression. The same attractor algorithm can be applied for other types of mutually correlated features, rather than genes. Therefore, more generally, the term “metagene” is an example of a “metafeature”, and it has also been implemented in MATLAB’s *metafeatures* function in the Bioinformatics Toolbox.

Using every available gene as seed identifies a limited number of strong co-expression signatures, each resulting in identical form from numerous seed genes. For methylation data, due to the excessively high number of methylation probes, we used a heuristic procedure for the exhaustive search to reduce computational complexity. The procedure, together with additional selection and filtering criteria for the validity of converged signatures to represent significant biological events (such as having a sufficient number of genes with high scores in each of them) are detailed in www.synapse.org/#!Synapse:syn5909000.

#### Probe-selection version for data sets with multiple probes for same gene

Different platforms have different probe designs and sometimes each gene may have multiple measurements at different probes, which are often highly correlated with each other, rather than representing independent gene isoforms. This can create a bias of favoring genes with multiple probes. To avoid this kind of bias, we analyzed the gene expression and methylation data sets by using a “probe-selection” version of the attractor-finding algorithm. As in the general algorithm, the probe-selection algorithm computes the association between the metafeature and all the available probes. It has an additional step, however, in which for each feature it only selects one probe having the highest weight. Only those probes are used for updating the metafeature for the next iteration. Due to the nature of the probe-selection algorithm, probes not associated with unique genes are ignored.

#### Consensus version for multiple data sets

To identify common signatures shared across multiple diseases, we used a “consensus” version of the attractor-finding algorithm, which simultaneously takes into account all individual data sets. In each iteration for generating a new metafeature, the association measure of each feature with the immediately preceding metafeature is evaluated as the weighted median of the corresponding association measures taken from the individual data sets. The weights are proportional to the number of samples included in each individual data set. In one particular case, because the sample size of one methylation data set (GSE74193) is one magnitude higher than those of the other methylation data sets, we divided it into smaller subsets based on samples’ processing plates, resulting in eleven individual methylation data sets used in final consensus analysis.

Both the above probe-selection and consensus methods of the attractor-finding algorithm are also detailed in www.synapse.org/#!Synapse:syn5909000.

#### Filtering consensus signatures by analyzing their presence in individual data sets

After identifying the consensus signatures, for each data set, we used the average value (expression or methylation) of the top ten probes of all the consensus signatures as seeds to run the probe-selection attractor-finding algorithm, thus deriving the particular individual versions of each signature. Then, we evaluated the pairwise overlap between each individual signature and each consensus signature in terms of gene symbols using the hypergeometric test (one-tailed version of Fisher’s exact test). We accepted the presence of a consensus signature in the individual data set if its overlap with the individual signature that it derived was the most significant (i.e., with the smallest *P* value) compared with its overlaps with other individual signatures of this data set and had *P* value less than 0.05. After obtaining the results for all the individual data sets, we removed from the final list any consensus signatures that were not present in the majority (i.e., more than half) of the individual data sets.

### Statistical analysis

As described above, each attractor signature defines a ranked set of genes along with selected probes depending on their scores. The average values of the top ten probes were used to represent the levels of corresponding signatures. For DNA methylation profiles, we transformed the methylation to *M* values as recommended^32^ and then took the average.

#### Cell type specificity analysis

The significance of cell type enrichment was assessed with the hypergeometric test by comparing the cell type markers and the genes for which the mapped probes have scores higher than 0.5 in the signature. To correct for multiple testing, we adjusted the resulting *P* values with the false discovery rate (FDR) method using the *p.adjust* R function with parameter *method* *=* *“fdr”*. A signature is considered to be enriched for one specific cell type if it has significant overlap (*P* < 0.05) with each reference list of markers.

#### Functional enrichment analysis

We used the Molecular Signatures Database (MSigDB) with the gene set enrichment analysis (GSEA) software^33^ (v6.2) to explore the biological functions or processes overrepresented in specific gene sets, such as the identified co-expression and co-methylation signatures. The MSigDB database contains eight major annotated gene set collections, including Gene Ontology (GO) gene sets, hallmark gene sets, etc. It outputs the hypergeometric *P* value and the FDR *q* value according to the Benjamini–Hochberg procedure as an estimate of statistical significance for the overlap with these gene sets. To provide evidence of translational impact, we used the STRING^34^ database (v10.5) to investigate the protein–protein interactions (PPIs) and protein functional analysis within each signature. For each signature, we used genes with scores higher than 0.5 as inputs, and limited their size to 500. FDR *q* values < 0.05 were considered significant.

#### Association identification

To investigate the association of the signatures with disease diagnosis, we used the linear mixed-effects (LME) model to evaluate the significance of disease association for each type of disorder. Since there are multiple data cohorts of the same disorder included, we used a random effect of study to consider the inter-study variability. To account for potential confounding effects, we evaluated results derived from two LME models, as follows:

$${\mathrm{Model}\,{\mathrm{0:Signature}\sim {\mathrm{Diagnosis}} + \left( {1|{\mathrm{Study}}} \right)}},$$

$${\mathrm{Model}}\,{\mathrm{1:Signature}\sim {\mathrm{Diagnosis}} + {\mathrm{Age} + {\mathrm{Gender}} + {\mathrm{PMI}} + \left( {1|{\mathrm{Study}}} \right)}}.$$

In *Model 0*, we obtained a “pure” significance of disease association without including other covariates, and in *Model 1*, we obtained a “confounder-adjusted” disease significance by treating age, gender, and postmortem interval (PMI), the covariates which are available in most of the data sets, as fixed effects. We used the *lmer* function implemented in the *lme4* R package^35^ to fit the model using restricted maximum likelihood (REML, the default in *lme4*), and derived *P* values by the Satterthwaite’s degrees of freedom method with the *lmerTest* R package, as suggested for producing acceptable Type I error even for smaller sample sizes^36^. We further took into account the potential confounding effects of other covariates (antipsychotics dose, method of death, substance abuse, and smoking) which are only available in some data sets. We assessed their association with corresponding signatures separately in individual data set by performing one-way analysis of variance. We also performed the Mann–Whitney *U* test, a nonparametric approach, to evaluate the disease association for each disease in the individual data sets to confirm the results. In all cases above, the threshold of statistical significance for *P* values was set to 0.05.

The correlation between same-type signatures (i.e., expression to expression, methylation to methylation) was examined using the Pearson’s correlation test. The “expression–methylation” (“E–M”) correlation between one co-expression signature and one co-methylation signature cannot be directly evaluated by using the same method, because there are no expression and methylation data coming from identical samples. As an alternative, we evaluated the E–M interrelationship through gene membership comparison by taking a significant overlap as indication that indeed methylation of the intersection genes affects the expression of those genes. We used the hypergeometric test to evaluate the significance of the overlap between the mapped gene symbols of the two signatures, for which the total gene pool was the intersection of the genes included in both expression and methylation platforms. The resulting *P* values were adjusted by the FDR method. Similarly, all *P* values for measuring the significance of overlaps in the context were evaluated using hypergeometric test.

## Results

The consensus attractor finding (Materials and methods) of the combined data cohorts identified three consensus co-expression signatures and five consensus co-methylation signatures, to which we refer as E1 through E3 and M1 through M5, respectively. The top-ranked probes and the mapped gene symbols of each signature are listed in Table 2. A more complete description of all the signatures can be found in Data Table S1.

**Table 2:** Top probes in each PFC consensus signature. There are three columns shown for each signature, which denote the probe ID, gene symbols, and scores for the top 15 probes. (a) Consensus co-expression signatures, E1–E3. (b) Consensus co-methylation signatures, M1–M5

(a)								
E1				E2			E3	
Probes	Genes	Scores	Probes	Genes	Scores	Probes	Genes	Scores
209300_s_at	NECAP1	0.8463	202800_at	SLC1A3	0.8772	209769_s_at	SEPT5-GP1BB	0.8030
212990_at	SYNJ1	0.8265	207761_s_at	METTL7A	0.8247	217696_at	FUT7	0.7826
202854_at	HPRT1	0.8228	203908_at	SLC4A4	0.8194	214122_at	PDLIM7	0.7676
208841_s_at	G3BP2	0.8092	202936_s_at	SOX9	0.8180	216940_x_at	YBX1	0.7666
213745_at	ATRNL1	0.8031	201667_at	GJA1	0.8092	214105_at	SOCS3	0.7537
204552_at	INPP4A	0.8022	212230_at	PPAP2B	0.7954	209979_at	ADARB1	0.7524
201889_at	FAM3C	0.8009	201876_at	PON2	0.7926	209730_at	SEMA3F	0.7439
205352_at	SERPINI1	0.8005	212377_s_at	NOTCH2	0.7898	216680_s_at	EPHB4	0.7425
205280_at	GLRB	0.7963	203296_s_at	ATP1A2	0.7888	207306_at	TCF15	0.7417
209274_s_at	ISCA1	0.7942	206465_at	ACSBG1	0.7684	216345_at	ZSWIM8	0.7385
211763_s_at	UBE2B	0.7915	209210_s_at	FERMT2	0.7624	206824_at	CES1P1	0.7325
202670_at	MAP2K1	0.7909	221796_at	NTRK2	0.7613	216821_at	KRT8P11	0.7305
218042_at	COPS4	0.7866	212850_s_at	LRP4	0.7505	202828_s_at	MMP14	0.7244
221207_s_at	NBEA	0.7864	205328_at	CLDN10	0.7432	216076_at	L3MBTL1	0.7219
213423_x_at	TUSC3	0.7861	203120_at	TP53BP2	0.7412	205212_s_at	ACAP1	0.7214

(b)								
M1				M2			M3	
Probes	Genes	Scores	Probes	Genes	Scores	Probes	Genes	Scores
cg10717149	SLC25A14	0.9911	cg22655232	PPP1R2P9	0.9784	cg26765599	NMRAL1	0.9040
cg04317640	SLC16A2	0.9910	cg11049634	BCOR	0.9778	cg06081917	BFAR	0.9013
cg16221895	EDA	0.9908	cg05130312	LOC286467	0.9740	cg17032990	MAP4K4	0.8983
cg14191108	MAOA	0.9907	cg14372935	PIR	0.9722	cg02473439	CCAR1	0.8979
cg10981178	ZBTB33	0.9899	cg06780606	EDA	0.9716	cg02193425	FAM50B	0.8972
cg26505478	CUL4B	0.9893	cg09791535	GPC4	0.9714	cg02313013	TMCC3	0.8942
cg23696472	TSPYL2	0.9892	cg09192294	LAS1L	0.9701	cg00489902	POLE	0.8926
cg05806018	AFF2	0.9890	cg07801607	ZMAT1	0.9696	cg10521450	SH3PXD2A	0.8922
cg11594566	LINC00086	0.9884	cg04690567	PHF8	0.9683	cg02041593	SEMA5B	0.8917
cg10201390	DYNLT3	0.9882	cg00098732	HS6ST2	0.9662	cg00245075	GALNT6	0.8911
cg20749341	LONRF3	0.9881	cg12653510	XIST	0.9662	cg19885979	TRIM26	0.8902
cg22164912	GNL3L	0.9877	cg27551771	KIAA1210	0.9639	cg00452755	RCC1	0.8900
cg20766178	NHSL2	0.9875	cg01037726	PNCK	0.9627	cg02450267	MOG	0.8876
cg18989810	DUSP9	0.9874	cg04704683	POF1B	0.9617	cg23384027	NFE2	0.8875
cg22604777	MAGEH1	0.9874	cg08209935	ARMCX5	0.9602	cg02713352	B4GALNT1	0.8874

M4			M5					
Probes	Genes	Scores	Probes	Genes	Scores			
cg12268888	FAM198A	0.8628	cg12547839	UBE2O	0.8539			
cg09063372	HDGF	0.8506	cg22330763	SLC29A1	0.8421			
cg11150308	SRP68	0.8505	cg04101806	AFF3	0.8203			
cg16129988	UQCRC1	0.8468	cg23400122	MSRA	0.8179			
cg11371394	TGFBRAP1	0.8406	cg26218110	BAHCC1	0.8153			
cg04233747	PRELID2	0.8403	cg25119743	CELF2	0.8023			
cg03330867	TELO2	0.8398	cg06372223	SLC7A5	0.8023			
cg04426297	B3GAT3	0.8394	cg24897320	CYB561D1	0.8008			
cg24695828	ZNF566	0.8324	cg14706739	DMTN	0.7987			
cg01923255	ATG14	0.8321	cg08202720	PER2	0.7983			
cg26897054	DEDD2	0.8317	cg17518776	PACSIN1	0.7970			
cg11111696	ZNF438	0.8312	cg20318252	MSI2	0.7966			
cg25426560	DHX16	0.8309	cg24107728	LRP8	0.7962			
cg05623562	RBFA	0.8300	cg08506743	NTM	0.7943			
cg24715473	CNEP1R1	0.8290	cg20685981	MEGF8	0.7929			

**Fig. 1:**
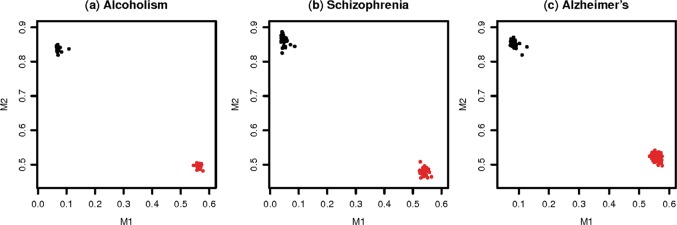
Gender-related PFC consensus co-methylation signatures (M1 and M2) in different disorders. The *x*-axis and *y-*axis show the methylation levels of M1 and M2 signatures, respectively, which were calculated as the average *β* values of the top ten CpG sites in each signature. Female and male samples are represented in red and black colors, respectively. The plots are labeled with the associated neuropsychiatric disorders. **a** Alcohol use disorder, of data set GSE49393. **b** Schizophrenia, of data set GSE74193 in processing plate “Lieber_289”. **c** Alzheimer’s disease, of data set GSE80970

Taking into consideration the gender-related differences in gene expression and methylation, including the fact that there are such differences in neuropsychiatric disorders^37^, we first investigated if any signature is related to gender. Among the co-methylation signatures, we found that M1 and M2 are purely gender-related (due to the presence of both genders in the data) (Fig. 1), consistent with the fact that their top-ranked genes are almost exclusively located in sex chromosomes. For example, XIST, one of the top genes of signature M2 (Table 2b), plays a critical role in the process of X-chromosome inactivation in mammalian females, an early developmental mechanism through which one of the X chromosomes is silenced by the combination of DNA methylation and histone modifications to provide dosage compensation^38^. Since they are otherwise unrelated with disease phenotypes, we do not include them in the following analyses.

Co-expression and co-methylation signatures include contributions from several distinct cell subpopulations in the heterogeneous brain tissues, from cell types such as neurons, astrocytes, oligodendrocytes, and immune cells, which can be further decomposed into cell subtypes. Because their corresponding probes have the property that their expression or methylation values tend to have higher or lower values in concordance, it is likely that some of these signatures reflect the relative abundance of such a subpopulation, which varies from sample to sample. Alternatively, co-expression or co-methylation may be due to the varying activation of a particular mechanism within the same subpopulation, as may result, e.g., when the expression levels of multiple genes are affected by their simultaneous methylation.

Therefore, we investigated which among the consensus signatures we found are predominantly due to the varying abundances of particular cell types across the samples. To identify the enrichment of the consensus signatures in cell-type-specific genes, we made use of two published gene lists of such gene markers derived from single cell analyses for reference^39,40^ (Materials and methods). The first reference list is taken from a study providing a classification of human brain cells into six major types^39^. The second reference list comes from a study providing a full list of marker genes of nine cell types found in the mouse cortex^40^. Although not resulting from human tissues, these extensive and detailed listings from mice are useful for additional scrutiny and validation. The results of enrichment analysis of cell type markers using the two lists were highly consistent (Data Table S2). All of the signatures were found enriched for some specific cell types except for E3 and M4.

For the co-expression signatures, this analysis revealed that signature E1 is enriched for neuronal markers (*P* = 0.0033 using the human markers). As the mouse cortex gene set contains particular neuronal subtypes, we further found that E1 has the highest overrepresentation of markers for the interneuron subtype (*P* = 0.0018). On the other hand, we found that signature E2 is highly enriched for astrocyte-specific markers (such as SOX9, GJA1 ranked at the top) in both gene lists (*P* = 2.2 × 10^−13^ using the human markers and *P* = 1.4 × 10^−16^ using the mouse markers).

**Fig. 2:**
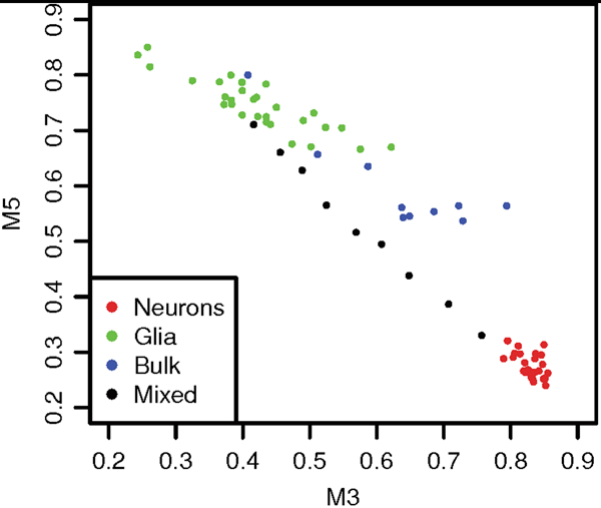
**Negative correlation between cell-type-specific PFC consensus co-methylation signatures (M3 and M5) in different cell populations.** The *x*-axis and *y*-axis show the methylation levels of the M3 and M5 signatures, respectively, which were calculated as the average *β* values of the top ten CpG sites in each signature. The samples come from the data set GSE41826, which includes methylation data of separated neurons (red), separated glia (green), as well as mixed (black for manually mixed and blue for bulk samples) from healthy human PFC tissues. The proportion of neurons to nonneurons in the empirically mixed samples range from 10 to 90% in 10% increments

For the co-methylation signatures, we found that signatures M3 and M5 are enriched for markers of glia and neurons, respectively (Data Table S2), and they were strongly and negatively correlated with each other (Pearson’s *r* < −0.75, *P* < 1.2 × 10^−8^; Data Table S3). We validated this finding by checking the methylation levels of M3 and M5 in an independent human PFC methylation data set of isolated neurons and nonneurons as well as manually mixed and bulk samples^41^ (GSE41826) (Fig. 2). Moreover, M3 was found associated with the co-expression signature E2 (*P* = 0.010; Materials and methods), which is consistent with the aforementioned facts that E2 is overrepresented in astrocyte markers and M3 is enriched for different subtypes of glial cell including astrocytes. Taken together, these findings indicate that M3 and M5 reflect the relative abundances of neurons vs. glial cells in samples, which are negatively associated, by including particular hyper- and hypo-methylated loci in these two subpopulations.

We evaluated the signatures’ associations with disease diagnosis considering potential confounding effects (Materials and methods; Supplementary Notes). As a result, we observed that the co-expression signatures E1 and E2 showed concordantly significant associations in more than one type of neuropsychiatric disorders (Data Table S4), and these associations are unlikely to be spurious due to confounding factors. Moreover, both signatures were enriched for PPI networks (*P* < 1.0 × 10^−16^ for E1, *P* = 8.4 × 10^−14^ for E2). Specifically, E1 was found broadly downregulated in several types of diseases, a topic discussed in detail in the next section.

The other signature, E2 was found upregulated in schizophrenia (*P* = 1.8 × 10^−3^) and bipolar disorder (*P* = 2.1 × 10^−3^). One of the GSEA top hits suggested that E2 is also enriched for genes which were found upregulated in brains with Alzheimer’s disease^42^ (FDR *q* value = 1.8 × 10^−19^). Finally, we observed a substantial overlap between E2 and an astrocyte-related co-expression module (named “CD4”) that was recently found positively associated with multiple psychiatric disorders^43^ (*P* = 1.1 × 10^−114^). Functional annotation analysis indicated the enrichment for biological pathways related to nervous system development and glial cell differentiation.

## The E1–M4 pair indicates a disease-related stress-induced epigenetic mechanism

The most significant E–M association was observed between signatures E1 and M4 (*P* = 1.9 × 10^−4^; Data Table S3), which share a large proportion of genes in common.

We first discuss the co-expression signature E1, which we found to be the one most significantly associated with disease diagnosis among all the identified consensus signatures. We observed significant downregulation of E1 in the presence of disease in several types of neuropsychiatric disorders except for depression and alcohol use disorder, and we confirmed this striking association using validation data sets (Table 3). We took into account the potential confounding effects of all available traits (including age, gender, manner of death, substance abuse, antipsychotic treatment usage, etc.), and our results suggest that the significant disease association we identified in signature E1 may not be confounded by these factors (Materials and methods; Supplementary Notes).

**Table 3:** Disease association of co-expression signature E1. Shown are the *P* values of association with diagnosis in different disorders resulting from the LME models (Materials and methods) for signature E1. Columns annotated by “*Model 0*” and “*Model 1*”, represent the “pure” and “confounder-adjusted” cases, respectively. Full results for other signatures can be found in Data Table S4

(a) Discovery data		
Diseases	E1 (Model 0)	E1 (Model 1)
Schizophrenia	1.7 × 10^−3^	1.1 × 10^−3^
Bipolar disorder	3.8 × 10^−5^	3.3 × 10^−5^
Parkinson’s	0.013	0.040
Major depression	0.19	0.13

(b) Validation data		
Diseases	E1 (Model 0)	E1 (Model 1)
Schizophrenia	0.011	0.011
Bipolar disorder	0.014	0.014
Parkinson’s	7.1 × 10^−4^	0.016
Alzheimer’s	0.018	0.026
Alcohol use disorder	0.99	0.99
Major depression	0.92	0.89

The disease-associated downregulation of signature E1 is supported by its large overlap (using the genes for which the scores in E1 are higher than 0.5) with previously identified differentially expressed gene sets in different illnesses. For instance, E1 genes are overrepresented in a set of genes that were identified downregulated in the PFC of patients with schizophrenia^44^ (*P* = 9.1 × 10^−7^). Furthermore, the top GSEA result for E1 revealed significant enrichment for genes downregulated in the brain from patients with Alzheimer’s disease^42^ (FDR *q* value = 3.0 × 10^−^^317^). Finally, many common genes were found included in both E1 and a neuronal module (named “CD13”) that had been found downregulated in multiple psychiatric illnesses^43^ (*P* = 2.3 × 10^−^^40^). These findings strongly suggest that the downregulation of the consensus co-expression signature E1 represents an important biological event occurring in the brain affecting different neuropsychiatric disorders.

As previous studies indicated^42,44^, and in concordance with the GSEA results of signature E1, some of the downregulated genes are involved in biological processes related to neuronal functions including neurotransmitter transport, signaling pathways and various energy metabolism processes. On the other hand, as mentioned previously, E1 was found enriched in neuronal markers, particularly of the interneuron subtype, which allows for a possibility that the disease correlation of E1 is caused by the variances of this particular cell population, as was the case for signature E2. To elucidate in what ways E1 is related to interneurons and associated with disease, we designed an experiment as described below.

In the experiment, we made use of the set of interneuron density markers whose expression levels were identified to be significantly and positively correlated with the density of CALB1-positive GABAergic interneurons^45^, which also appeared as the second top GSEA hit for signature E1 (FDR *q* value = 5.9 × 10^−250^). The null hypothesis was that the downregulation of E1 observed in patients with disease is caused by the decreasing population of interneurons, in which case we should expect to see that the disease association becomes reduced when we remove from the E1 gene list the markers for interneuron density. Therefore, we compared the disease association of the subset of E1 genes without those interneuron markers, referred to as “Set E1/Interneuron” with that of the E1 signature. As a result, the disease association for the Set E1/Interneuron became stronger in most cases, rejecting the null hypothesis (Table 4, see columns named “E1” and “E1/Interneuron”). This suggests that the strong disease association of signature E1 in various neuropsychiatric disorders is not caused by the allocation of interneuron subpopulation in samples.

**Table 4:** Strengthened disease association compared with E1 alone. Shown are the confounder-adjusted *P* values of association with diagnosis in different disorders for three E1-related gene sets in the discovery data. Columns “E1”, “E1/Interneuron”, and “E1 ∩ M4” represent the cases for E1 signature alone, the subset of E1 genes without GABAergic interneuron markers, and the intersection of genes included in both E1 and M4

Diseases	E1	E1/interneuron	E1∩M4
Schizophrenia	1.1 × 10^−3^	4.6 × 10^−4^	2.5 × 10^−4^
Bipolar disorder	3.3 × 10^−5^	1.1 × 10^−5^	2.7 × 10^−6^
Parkinson’s	0.040	0.086	0.032
Major depression	0.13	0.12	0.050

We then looked at the significant overlap of genes found between the co-expression signature E1 and the co-methylation signature M4, which implies an underlying epigenetic regulation mechanism. We selected the overlapping genes between the E1 and M4 signatures (using genes with scores > 0.5), referred to as “Set E1∩M4”, and ranked them by the minimum of their scores in E1 and M4 gene lists so that the top gene has the highest minimum score (the ranked list of the top 15 genes of Set E1∩M4 is shown in Table 5, while the full list can be found in Data Table S5). We evaluated the disease association of Set E1∩M4 using the average expression values of the top-ranked ten genes in the set and compared with the case of E1 itself. As a result, we observed overall enhancement of the association with diagnosis in the cases of the Set E1∩M4 (Table 4, columns labeled “E1” and “E1∩M4”). This result suggests that the co-methylation signature M4 contributes to refining the co-expression signature E1 with respect to the association with disease diagnosis through an epigenetic mechanism.

**Table 5:** Top-ranked genes in the Set E1∩M4. This table shows the top 15 overlapping genes between signatures E1 and M4, ranked by the minimum of their scores in the two signature gene lists. The four columns represent the gene symbols, corresponding probe IDs in E1 and M4 signatures, and the minimum scores, respectively

Gene symbols	Probes in E1	Probes in M4	Min scores
CAND1	208838_at	cg17524854	0.7499
DYNC1LI1	217976_s_at	cg25390230	0.7253
ATP5A1	213738_s_at	cg10619144	0.6912
EFR3A	212150_at	cg09396107	0.6786
MEAF6	218165_at	cg03112782	0.6774
PNMA1	218224_at	cg23681213	0.6772
SEC23A	212887_at	cg02056847	0.676
ZNHIT3	212544_at	cg09922935	0.6697
PPP2R5C	201877_s_at	cg08393828	0.6676
NDUFAB1	202077_at	cg21989500	0.6675
EIF1B	201738_at	cg25839330	0.6672
PPP3CA	202429_s_at	cg00302793	0.6667
SLC30A9	202614_at	cg09414773	0.6599
UQCRC2	212600_s_at	cg03031583	0.6595
RGS7	206290_s_at	cg24472496	0.6592

Furthermore, regarding the co-methylation signature M4 itself, we found that it has a unique attribute among all the nongender-related consensus co-methylation signatures, in that it contains a remarkably high proportion of methylation probes located at promoter-associated regions (*P* = 8.0 × 10^−149^) and CpG islands (*P* = 0) when compared with the overall sites for the methylation array (Data Table S6), implicating its function of epigenetic regulation.

To understand the biological functions represented by the E1–M4 signature, we applied functional annotation analysis on the intersection genes. First, significant enrichment of PPIs (*P* = 2.2 × 10^−16^) indicates the meaningful biological connections and regulatory functions among the proteins encoded by these genes. The small GTPase superfamily was the top hit by assessing the overlap with InterPro protein domains and features database^46^. Among the results of GSEA analysis, the set of genes downregulated in brains with Alzheimer’s disease^42^ remains at the top (FDR *q* value = 9.7 × 10^−^^115^). Overrepresentation was also found in sets of genes having at least one occurrence of highly conserved motifs matching binding sites for transcription factors SP1 (FDR *q* value = 3.1 × 10^−25^) and LEF1 (FDR *q* value = 5.6 × 10^−^^14^), which may provide hints about the nature of the underlying epigenetic mechanism. Moreover, we found that stress-related biological processes (GO) were enriched in the genes of E1–M4 signature (FDR *q* value < 10^−6^). We further confirmed that these stress-related gene sets were also overrepresented in the respective E1 and M4 signatures, but not in any of the other consensus signatures (Data Table S7).

Along with the significant E–M interrelationship and strong disease association, these findings collectively suggest that the E1–M4 signature pair represents some stress-induced epigenetic mechanism, which could be associated with the underlying etiology of several neuropsychiatric disorders.

## Discussion

To investigate the underlying pathological mechanism(s) common to various neuropsychiatric diseases, we did meta-analyses on the expression and methylation data of postmortem PFC samples collected from patients with six different neuropsychiatric disorders along with healthy controls (Table 1). By using our unsupervised approach, we identified several consensus co-expression and co-methylation signatures present in similar forms across different data sets and diseases (Table 2). By scrutinizing these signatures’ disease associations and interrelationships, our study revealed some biological abnormalities strongly associated with disease diagnosis.

For example, we identified an astrocyte-related co-expression signature, E2, which was observed upregulated in patients with schizophrenia and bipolar disorder, and functional enrichment analysis also indicated its overlap with genes found to be upregulated in Alzheimer’s disease. Previous studies have suggested that the changes in expression of astrocyte markers could be linked to neuroinflammation in these diseases^47,48^. We did not observe such positive association in other disorders. Taking the major depressive disorder as an example, our results showed that, on the contrary, E2 is negatively, though not very significantly, associated with disease diagnosis (Data Table S4). Indeed, studies have reported persistent decreases in astrocyte-specific markers in patients with major depression^49^, indicating disease association with decreased density or hypofunction of astrocytes, and there is also experimental evidence provided for understanding the underlying pathogenic mechanism using animal models^50^.

Our work resulted in the derivation of several co-expression and co-methylation signatures using an algorithm designed to point to the core of the underlying mechanisms, which suggests that the top genes of such signatures are more biologically accurate compared with traditional clustering methods. However, the main feature of our study is the examination of the interrelationships between such expression and methylation signatures in search of epigenetic mechanisms. Understanding disease-associated epigenetic mechanisms may provide opportunities of developing novel therapeutic options.

Using this approach, our main finding was the novel discovery of a significant interrelationship between the co-expression signature E1 and co-methylation signature M4, which indicates an epigenetic relationship. On the one hand, the signature E1 is enriched for interneuron markers and we provided evidence that the derivation of the signature is due to a variation of the expression levels of such interneurons, rather than a varying abundance of their subpopulation. On the other hand, E1 is found strongly down regulated in multiple types of neuropsychiatric diseases (Table 3). The disease association becomes further strengthened when we consider the shared genes between E1 and M4 (Table 4), suggesting that this sharper disease-associated signature is related to an epigenetic mechanism involving the E1–M4 genes (Table 5). The overrepresentation of promoter-associated regions and CpG islands in the corresponding genes of M4 also complements its role of epigenetic regulation. Future experimental research on those genes has the potential of uncovering the details of the biological mechanism underlying the epigenetic signature and leading to therapeutic applications.

We did not, however, observe a significant disease association of the signature in major depressive disorder and in alcohol use disorder, suggesting that their diagnosis is often independent of the underlying biological mechanism. This is consistent with the fact that there is comorbidity between depression and alcohol use disorder^51^. Such differences reflect the heterogeneity of neuropsychiatric disorders. For example, major depressive disorder is known to have small heritability compared with other disorders^52^.

In addition, functional annotation analysis of the E1–M4 genes revealed the enrichment of stress-related biological processes. While the genes highlighted under the identified GO terms may reflect internal cellular processes, most gene activities are attributed to pathways involving exogenous stressors. Notably, stress response and related epigenetic regulation mechanisms in the brain have been investigated and implicated in neuropsychiatric diseases^8,9,53,54^, bringing up the possibility that these effects are driven by psychiatry-relevant psychosocial stressors or other relevant biological processes and should be investigated in future studies.

Our findings should be interpreted in light of some caveats. Although we have taken care of controlling for all known confounders, unmeasured variables specific to disease states may influence the results to some degree in epigenomic and transcriptomic studies. The identified mechanisms in this study may well be causal and important underlying features to disease etiology, but there is also a chance that this is not true.

In summary, our integrative data mining and analysis of some of the identified consensus co-expression and co-methylation signatures suggest the presence of a stress-related epigenetic mechanism associated with different neuropsychiatric diseases.

## Supplementary information

SI summary

Supplementary notes

Data Table S1

Data Table S2

Data Table S3

Data Table S4

Data Table S5

Data Table S6

Data Table S7
