# Supplementary material for: Meta-analysis of expression and methylation signatures indicates a stress-related epigenetic mechanism in multiple neuropsychiatric disorders
Source: Transl Psychiatry. 2019 Jan 22;9:32. doi: 10.1038/s41398-018-0358-5 (PMC6342918; doi:10.1038/s41398-018-0358-5)
Supplement: Supplementary file 1 — SI summary [file 41398_2018_358_MOESM1_ESM.pdf]

# Summary of Supplementary Information

**Supplementary Notes (.PDF)**

Complementary information for confirming the significant disease associations of signatures we claimed in the main text.

**Data Table S1 | Consensus PFC signatures (.XLSX)**

Description of all the consensus co-expression (the sheet named "cons_exprs_signatures") and co-methylation (the sheet named "cons_methyl_signatures") signatures. For each signature, we listed the probe names, gene symbols and scores (rounded to four decimal places) of probes with scores higher than 0.5, and the size is limited to 500.

**Data Table S2 | Cell type enrichment (.XLSX)**

*P* values of the enrichment for different cell type markers, using the single cell reference lists from human (the sheet, named "human_sc_markers") and mouse (the sheet named "mouse_sc_markers") brain tissues.

**Data Table S3 | Pairwise correlation of signatures (.XLSX)**

The first two sheets, with names as "exprs_exprs" and "methyl_methyl", show the *P* values and Pearson's r values of pairwise correlations between the same-type signatures. The third sheet, named as "exprs_methyl", shows the *P* values of hybrid correlations between co-expression and co-methylation signatures.

**Data Table S4 | Disease association by LME (.XLSX)**

Two versions of significance of disease association for each signature evaluated by the linear mixed-effects regression method are included, of which one is "pure" (*Model 0*, without covariates included) and the other is "confounder-adjusted" (*Model 1*, with covariates included). The two sheets contain the results for co-expression and co-methylation signatures, respectively.

**Data Table S5 | E1-M4 signature (.XLSX)**

Full list of the overlapping genes between the E1 and M4 signatures. The columns represent the gene symbols, the corresponding probes, the scores in the two signature gene lists and the minimum of the two scores. The list is ranked by the minimum scores in decreasing order.

**Data Table S6 | Methylation signatures enriched regions (.XLSX)**

*P* values evaluated for the enrichment of CpG methylation probes located in specific genomic regions for all the consensus co-methylation signatures.

**Data Table S7 | Significance of GO STRESS (.XLSX)**

The first sheet, named "overall_p_value", contains the information of the number of overlapping genes and the corresponding *P* values for each consensus signature with the two stress-related gene lists: GO_CELLULAR_RESPONSE_TO_STRESS, GO_REGULATION_OF_RESPONSE_TO_STRESS. The next two sheets, named "overlapping_genes_GO_CELLULAR" and "overlapping_genes_GO_REGULATION" explicitly list the overlapping genes with these two GO_STRESS gene lists for signatures E1, M4, and E1∩M4.
