# Supplementary material for: Meta-analysis of expression and methylation signatures indicates a stress-related epigenetic mechanism in multiple neuropsychiatric disorders
Source: Transl Psychiatry. 2019 Jan 22;9:32. doi: 10.1038/s41398-018-0358-5 (PMC6342918; doi:10.1038/s41398-018-0358-5)
Supplement: Supplementary file 2 — Supplementary notes [file 41398_2018_358_MOESM2_ESM.pdf]

Supplementary Notes for

Meta-analysis of expression and methylation signatures indicates a stress-related epigenetic mechanism in multiple neuropsychiatric disorders

Kaiyi Zhu, Tai-Hsien Ou Yang, Vincent Dorie, Tian Zheng, Dimitris Anastassiou*

*Corresponding author. Email: d.anastassiou@columbia.edu

I. Investigating potential confounding effects on the disease associations of E1 and E2 signatures from additional covariates.

In the main text, we identified significant and consistent disease associations for the co-expression signatures E1 and E2 by using linear mixed-effects (LME) models controlling for age, gender and postmortem interval. In addition to these three covariates, some additional ones were only available in some data sets, in which we evaluated their associations with the signatures per data set. We reasoned that, in any individual study, if the coefficients for potential confounders were not statistically significant while the coefficient for disease was, then the potential hidden bias introduced by not having these variables available in the full study would not be strong enough to explain away the observed, statistically significant association between signature and disease diagnosis.

Therefore, we analyzed the additional covariates' associations with E1 and E2 signatures in individual data sets by using the one-way ANOVA test (for "antipsychotics", we tested the association only on patients), and the resulting *P* values are listed in the tables. Related information was extracted from the original publications of the data sources.

A. SMRI_AltarC

Covariates	Suicide	Smoking	Alcohol	Drugs	Antipsychotics
E1	0.35	0.80	0.13	0.73	0.75
E2	0.45	0.68	0.77	0.82	0.46

B. SMRI_Bahn

Covariates	Suicide	Smoking	Alcohol	Drugs	Antipsychotics
E1	0.85	0.19	0.25	0.69	0.44
E2	0.29	0.28	0.68	0.70	0.75

C. Maycox *et al.*[1] [GSE17612]

Manner of death: The control and case samples in this data cohort have similar cause of death, bronchopneumonia being the most case followed by the carcinoma.

Substance abuse: The patients have no access to drugs or alcohol of abuse.
Antipsychotics: Most patients had been treated with neuroleptic drugs when they became available, with relatively low doses. Only one patient was neuroleptic naïve at death, and four patients took relatively high doses.

D. Narayan *et al.*[2] [GSE21138]

Covariates	Suicide	Antipsychotics
E1	0.45	0.09
E2	0.61	0.58

E. Ryan *et al.*[3] [GSE5388]

Covariates	Suicide	Alcohol	Drugs	Antipsychotics
E1	0.75	0.37	0.77	0.18
E2	0.85	0.78	0.72	0.16

F. Zhang *et al.*[4]; Zheng et al.[5] [GSE20168]

No more information is available.

G. Chang *et al.*[6]; Sibille et al.[7] [GSE54567, GSE54568, GSE54570]

Covariate	Method of death (natural, accidental, suicide)
E1	0.30
E2	0.52

Antipsychotics: Cases were free of psychotropic medication or illegal drugs, and controls were drug-free.

II. Replicating disease associations by nonparametric test.

In addition to the LME models, we also applied a robust nonparametric approach to obtain "baseline" significance of the disease association. It was performed per disease per data set. The results shown below confirm that significant associations with concordant directions occurred repeatedly in different data sets and disorders, from different studies.

The *P* values were calculated by performing the Mann-Whitney U test on the diagnosis in each individual data set. For data sets which contain more than one disease type, we tested the association for each disease separately. The arrows beside the *P* values indicate the direction of differential expression in patients: ↓ (↑) indicate that down- (up-) regulation of the signature was identified in patients.

(a) Discovery data sets

**Diseases**	**Data Sets**	**E1**	**E2**
Schizophrenia	SMRI_AltarC	NS	NS
	SMRI_Bahn	0.040 ↓	0.012 ↑
	GSE17612	NS	NS
	GSE21138	0.024 ↓	0.016 ↑
Bipolar disorder	SMRI_AltarC	1.9×10^{-4} ↓	NS
	SMRI_Bahn	0.035 ↓	NS
	GSE5388	0.015 ↓	NS
Parkinson's	GSE20168	0.016 ↓	NS
Major depression	SMRI_AltarC	0.016 ↓	NS
	GSE54567	NS	NS
	GSE54568	NS	NS
	GSE54570	NS	NS

NS, not significant, for *P* value ≥ 0.05.

(b) Validation data sets

**Diseases**	**Data Sets**	**E1**	**E2**
Alzheimer's	GSE36980	0.0040 ↓	NS
Alcohol use disorder	GSE49376	NS	0.036 ↑
Parkinson's	GSE68719	1.1×10^{-4} ↓	0.014 ↑
Major depression	GSE101521	NS	NS
Bipolar disorder	BrainGVEX	0.023 ↓	NS
Schizophrenia	BrainGVEX	0.0087 ↓	NS

NS, not significant, for *P* value ≥ 0.05.

References

1. Maycox PR, Kelly F, Taylor A, Bates S, Reid J, Logendra R *et al.* Analysis of gene expression in two large schizophrenia cohorts identifies multiple changes associated with nerve terminal function. *Mol Psychiatry* 2009; **14**(12)**:** 1083-1094.

2. Narayan S, Tang B, Head SR, Gilmartin TJ, Sutcliffe JG, Dean B *et al.* Molecular profiles of schizophrenia in the CNS at different stages of illness. *Brain Res* 2008; **1239:** 235-248.

3. Ryan MM, Lockstone HE, Huffaker SJ, Wayland MT, Webster MJ, Bahn S. Gene expression analysis of bipolar disorder reveals downregulation of the ubiquitin cycle and alterations in synaptic genes. *Mol Psychiatry* 2006; **11**(10)**:** 965-978.

4. Zhang Y, James M, Middleton FA, Davis RL. Transcriptional analysis of multiple brain regions in Parkinson's disease supports the involvement of specific protein processing, energy metabolism, and signaling pathways, and suggests novel disease mechanisms. *Am J Med Genet B Neuropsychiatr Genet* 2005; **137B**(1)**:** 5-16.

5. Zheng B, Liao Z, Locascio JJ, Lesniak KA, Roderick SS, Watt ML *et al.* PGC-1alpha, a potential therapeutic target for early intervention in Parkinson's disease. *Sci Transl Med* 2010; **2**(52)**:** 52ra73.

6. Chang LC, Jamain S, Lin CW, Rujescu D, Tseng GC, Sibille E. A conserved BDNF, glutamate- and GABA-enriched gene module related to human depression identified by coexpression meta-analysis and DNA variant genome-wide association studies. *PLoS One* 2014; **9**(3)**:** e90980.

7. Sibille E, Arango V, Galfalvy HC, Pavlidis P, Erraji-Benchekroun L, Ellis SP *et al.* Gene expression profiling of depression and suicide in human prefrontal cortex. *Neuropsychopharmacology* 2004; **29**(2)**:** 351-361.
